# Epidemiology, incidence and treatment of rectal cancer in young women case serie about 11 cases (case series)

**DOI:** 10.1016/j.amsu.2022.104693

**Published:** 2022-09-20

**Authors:** Amal Hajri, Amine Fatine, Yassine Eddaoudi, Saad Rifki El Jay, rachid Boufettal, Driss Erreguibi, Farid Chehab

**Affiliations:** Surgical Department of Cancerology and Liver Transplantation University Hospital Center Casablanca Morocco Faculty of Medicine and Pharmacy, Hassan II University, Casablanca, Morocco

**Keywords:** Rectal cancer, Young women, Epidemiology, Incidence, Abdominoperineal amputation, Case series

## Abstract

**Introduction:**

Rectal cancer constitutes, by its frequency and its gravity, a real concern of the public health in the world, it represents the eighth most frequent cancer. Its incidence is increasing in young people and in particular in women, in whom it remains a rare disease known for its poor prognosis.

The objective of our work is to highlight the epidemiological characteristics of rectal cancer in patients under 40 years of age, determine its incidence and outline the different therapeutic means.

**Materials and methods:**

Our work is a retrospective study with a descriptive aim on a series of 11 female patients aged less than 40 years, operated for rectal cancer in the department of digestive cancer surgery and liver transplantation Casablanca Morocco, over a period of 7 years from January 2013 to December 2019.

**Results:**

The average age of our patients was 34.8 years. The average diagnostic delay was 10 months. The most frequent clinical sign was rectorrhagia (90.9% of cases). On rectal examination, the tumor was inaccessible in 18.8% of cases and externalized in 9.09% of cases. It was located in the lower rectum in 36.36% of cases, the same for the middle rectum. Rectoscopy showed that the majority of tumors were circumferential (36.36%). The budding ulcerative aspect was the most frequently found with 7 cases or 63.63%. The histological study showed the predominance of lieberkühnian adenocarcinoma (63.63%). Thoracic-abdominal-pelvic CT scan showed liver metastases in only one patient (9.09%). Pelvic MRI showed invasion of the mesorectum in 5 cases (45.45%) and of the internal sphincter in 3 cases (27.27%). All our patients underwent laparotomy. Curative surgery was performed in 8 patients and 3 patients had palliative surgery. Preoperative radiotherapy was performed in 81.81% of cases. The evolution was marked by 27.27% of locoregional recurrences. The operative mortality was nil in our series.

**Conclusion:**

Detection of patients with precancerous conditions, screening for cancer in subjects at risk (familial recto-colic cancer, familial recto-colonic polyposis and ulcerative colitis), suspicion of cancer in the presence of any proctological sign, early diagnosis and curative surgical resection preceded by radiotherapy are the means that can improve the prognosis of rectal cancer in young women.

## Highlights

• Rectal cancer constitutes, by its frequency and its gravity, a real concern of the public health in the world.
• It represents the eighth most frequent cancer.
• Its incidence is increasing in young people and in particular in women, in whom it remains a rare disease known for its poor prognosis.

## 1 Introduction

Rectal cancer is a major public health concern worldwide due to its frequency and severity. On a global scale, it represents the eighth most frequent cancer. In young people, the incidence of rectal cancer has been increasing over the last few decades, particularly in young women, for whom it remains a rare disease with a poor prognosis. The occurrence of rectal cancer in young age can be caused by strong environmental carcinogens, weakened immunity, the presence of germline mutation (familial adenomatous polyposis FAP and Lynch syndrome) and inflammatory bowel diseases.

The objective of our work is to highlight, through the analysis of a series of 11 cases and a review of the literature, the epidemiological characteristics of rectal cancer in patients under 40 years of age, determine its incidence and outline the different therapeutic means. the work has been reported in line with the SCARE criteria [1].

## 2 Results and methods

The average age of our patients was 34.8 years. The average diagnostic delay was 10 months. The most frequent clinical sign was rectorrhagia (90.9% of cases) followed by rectal syndrome (81.81%) and then alteration of general condition and weight loss (72.72%). Transit disorders were found in 5 patients (45.45%), abdominal pain in the left iliac fossa in 1 patient (9.09%) and sub-occlusive syndrome in 1 patient (9.09%).

On rectal examination, the tumor was inaccessible in 18.8% of cases and externalized in 9.09% of cases. It was located in the lower rectum in 36.36% of cases, the same for the middle rectum. Rectoscopy showed that the majority of tumors were circumferential (36.36%).

The budding ulcerative aspect was the most frequent with 7 cases or 63.63% (Table 1). In only one case, the tumor was partially stenosing (9.09%). The histological study showed the predominance of lieberkühnian adenocarcinoma (63.63%) (Table 2).

**Table 1:** Tumor aspects.

Microscopic aspect	Number of cases	Percentage
lieberkühnian adenocarcinoma	7	63,63%
Mucosal colloid carcinoma	1	9,09%
Invasive carcinoma	2	18,18%
Squamous cell carcinoma	1	9,09%
Total	11	100%

**Table 2:** Histological type of the tumor.

Macroscopic aspect	Number of cases	Percentage
budding ulcerative	7	63,63%
Infiltrative	1	9,09%
budding ulcerative and infiltrative	1	9,09%
Vegetative	1	9,09%
Stenosing	1	9,09%
Total	11	100%

The tumors were predominantly well differentiated with a rate of 45.45%, those of medium and low differentiation represented each 9.09% of cases. Thoracic-abdominal-pelvic CT scan showed liver metastases in only one patient (9.09%), pulmonary parenchyma nodules in 2 patients (18.08%) and locoregional adenopathies in 3 patients (27.27%). Pelvic MRI showed invasion of the mesorectum in 5 cases (45.45%) and of the internal sphincter in 3 cases (27.27%). Carcinoembryonic antigen (CEA) and CA19-9 were measured in 9 patients, CEA was elevated in 1 case and CA 19–9 was elevated in 3 cases.

All our patients underwent laparotomy. Curative surgery was performed in 8 patients (abdominoperineal amputation with left definitive iliac colostomy (Fig. 2) in 4 cases and colorectal resection in the other 4, with colorectal anastomosis in 3 cases and coloanal anastomosis in 1 case) and 3 patients had palliative surgery (lateral sigmoidal colostomy on a rod), i.e. a resectable tumor rate of 72.7% (Fig. 3 and Fig. 4).

**Fig. 1:**
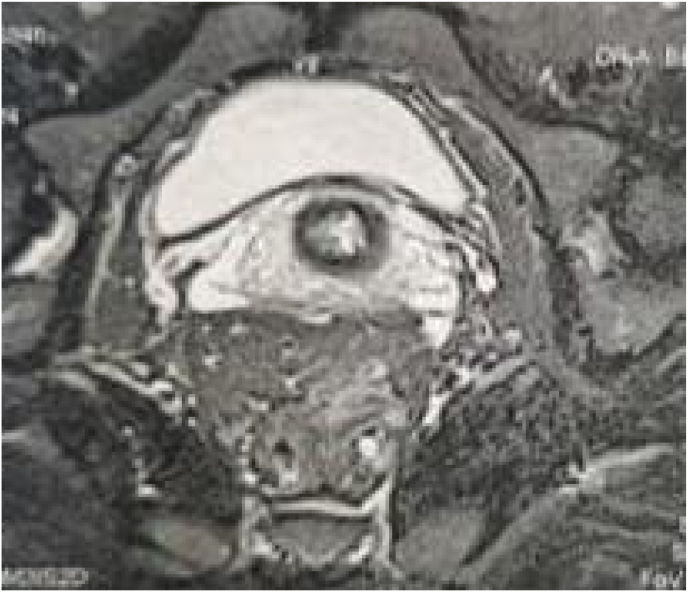
MRI of a patient showing a lower rectal tumor located 4 cm from the anal margin classified as T3N2Mx.

**Fig. 2:**
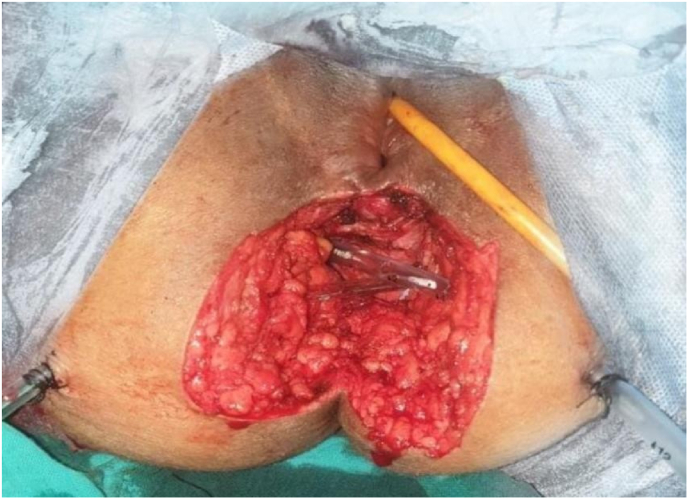
Intraoperative image of an abdominoperineal amputation with perineal drainage by two Redon drains.

**Fig. 3:**
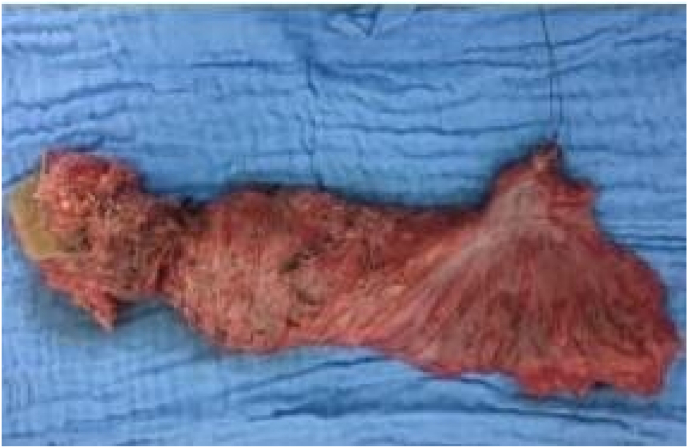
Surgical specimen of an abdomino-pelvic amputation with integrity of the mesorectum.

**Fig. 4:**
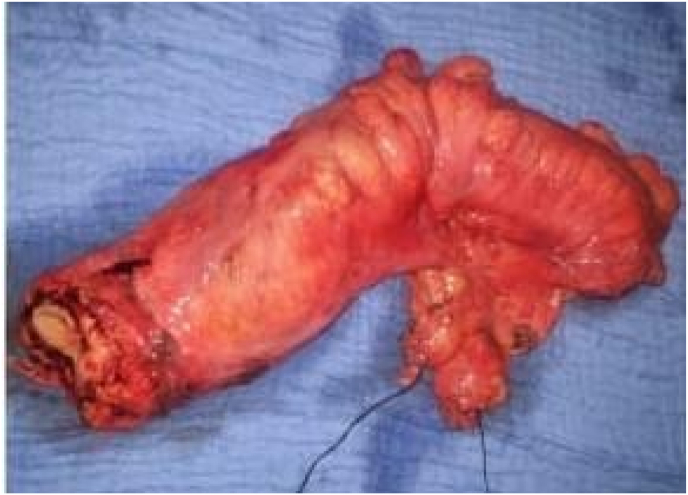
Colorectal resection specimen.

Preoperative radiotherapy was performed in 81.81% of cases. Immediate complications: 1 case of parietal infection. And one case of infection of the perineal wound, Long term complications: 1 case of recto vaginal fistula which was not cured given to the regression of the diameter of the fistula.

The evolution was marked by 27.27% of locoregional recurrences. The average time to occurrence of locoregional recurrence was 15 months (7–28 months). The operative mortality was 0% in our case serie.

## 3 Discussion

Colorectal cancer (CRC) ranks second in the world after breast cancer (22.9%) and before cervical cancer (8.8%) with an incidence of 571,204 new cases per year or 9.4% [1,2]. In the United States, colorectal cancer is the third most common cancer and the second most deadly [3]. The incidence of colorectal cancer has increased over the last few decades, with rates varying from one country to another [2,4,5]:

- High risk areas: Australian countries, North America, Western European countries, Japan.
- Intermediate risk areas: Eastern and Northern European countries.
- Low-risk areas: South America, Asia and Africa, Morocco.

This great disparity in the geographical distribution of cancers is probably due to environmental, dietary and other factors.

Colorectal cancer is rare in North African countries compared to Western countries. In Morocco, according to data from the cancer registry of the wilaya of Grand Casablanca, rectal cancer is the leading digestive cancer in women with an average of 46 new cases per year, 6 of which are under 40 years of age [6]. In Tunisia, colorectal cancer ranks first among all cancers. In central Tunisia, the standardized incidence of colorectal cancer is 6.1 per 100,000 inhabitants per year in women [7,8].

The precise incidence of rectal cancer remains difficult to determine insofar as most of the available studies treat colorectal cancers as a whole and because of the absence of in-depth epidemiological studies according to the age and sex of the patients.

Colorectal cancer occurs most often in elderly subjects. It is rare in young people. In the West, the incidence of colorectal cancer increases significantly between the ages of 40 and 45 years and continues to grow, doubling every 10 years [9]. However, in recent decades, the incidence of colorectal cancer has been increasing in younger people [10,11].

Diet, smoking, alcoholism, sedentary lifestyle and obesity increase the risk of developing CRC [12,13], and these are factors that affect the elderly much more because not only do habits change frequently over time but also their effects only become apparent after a long period of exposure [14,15]. However, predisposing factors such as inflammatory bowel disease (Crohn's, ulcerative colitis), familial polyposis, HPV infection and others are much more specific to the younger subject [14,16,17].

It is noted that the risk factors for CRC in the young subject are mainly genetic. Thus, the existence of a personal or family history, of precancerous lesions (PAF, HNPCC syndrome or IBD), or of a predisposing terrain should make one fear the appearance of rectal cancer. This is a fundamental element allowing the detection of high-risk subjects and thus offering the possibility of an early diagnosis through systematic screening.

In young subjects, the average diagnostic delay is often between less than one month and 6 months according to several series [[18], [19], [20]], which is much shorter than the delay found in our series. In general, symptoms may be underestimated or tolerated, especially in young patients who tend to wait until the symptoms become much more severe. The same applies to some physicians who, given the young age of the patients, do not suspect malignancy in the face of suggestive symptoms, especially since screening and surveillance in the absence of a particular family history do not concern this age group. Thus, because of the delay in diagnosis in young people, the patient is often very symptomatic at the time of diagnosis.

The most common and earliest functional sign of rectal cancer is rectorrhagia, which is secondary to tumor necrosis and ulceration [[21], [22], [23]], and may be isolated or associated with a rectal syndrome. It is a major warning sign requiring a rectal examination and systematic rectoscopy. Other signs frequently found are transit disorders, altered general condition, abdominal pain and sub-occlusive syndrome.

The positive diagnosis of rectal cancer is based on clinical, endoscopic and radiological investigations. The biopsy, performed with forceps, allows the diagnosis of certainty, which is necessary for the initiation of treatment [24].

Local rectal cancer assessment determines the extent of the tumor and plays an important role in determining neoadjuvant therapy. Rigid rectoscopy is the method of choice. Locoregional extent, including lymph node invasion, should be established by imaging. *Trans*-anal echo-endoscopy and pelvic MRI are the examinations of choice (Fig. 1). The workup must be completed by a thoracic-abdominal- pelvic CT scan to look for distant metastases. Tumor markers (carcinoembryonic antigen (CEA) and carbohydrate 19–9 antigen (CA 19–9)) constitute a prognostic element and are used for post-therapeutic surveillance of colorectal cancers to increase the chances of detecting recurrences at a stage where they are operable [5,25,26].

The main treatment is surgery, which aims to remove the tumor and its lymph node extensions, prevent loco-regional recurrence and ensure the longest and most comfortable survival. The quality of the surgical resection is the essential factor in the prognosis of rectal cancer. The modalities of rectal resection vary according to the site of the tumor, its possible extension to the neighboring organs, the patient's terrain, her desire for conservation and the state of the anal sphincter. Small T1N0 rectal tumors <3 cm can, under careful selection, be removed locally by *trans*-anal surgery or endoscopic excision [27].

The wide midline abdominal incision remains the most commonly used despite the development of laparoscopy. We note that there is no significant difference in terms of efficacy and morbidity rate between the laparoscopic procedure and the open procedure. In addition, postoperative complications and length of hospital stay are less after laparoscopy [28].

Curative surgical treatment can be conservative or non-conservative. Sphincter-conserving procedures involve partial or total resection of the rectum and mesorectum, and an anastomosis between the colon and the rectum or anal canal, thus avoiding a permanent colostomy for the patient. The anastomosis is sometimes protected by a temporary colostomy or ileostomy. The non-conservative treatment is represented by abdominoperineal amputation which has long been the reference treatment for cancers of the middle and lower rectum, it requires two approaches: abdominal and perineal and consists of the removal of the entire rectum, the levator ani muscles, the anal canal and its sphincter apparatus. A resection of the mesorectum and the fat of the ischio-rectal fossae is associated with it. In our series, the rate of abdominoperineal amputation was 36.36% which is close to the data of recent series in the literature [29].

Extra fascial excision of the mesorectum significantly reduces locoregional recurrence and respect for pelvic innervation limits the urinary and sexual sequelae of rectal resections. In the absence of damage to the fascia recti, preservation of the pelvic autonomic nerves allows an improvement in genitourinary results.

Measurement of the surgical margins (distal and circumferential) is essential to define the completeness of the resection and has a prognostic role. For upper third tumors, the mesorectal section should pass 5 cm below the lower tumor border. The distal safety margin (distance between the lower pole of the tumor and the distal rectal cut) should be ≥ 1 cm [30].

Node invasion is a major prognostic factor in rectal cancers. Thus, inferior mesenteric lymph node curing is warranted without tying the inferior mesenteric artery flush with the aorta. In case of a suspicious node in these territories, a sample will be taken for extemporaneous examination and a clip will be put in place for a later location.

Palliative surgery is essentially represented by palliative colostomy. Colonic prostheses may become a promising alternative to colostomy for lesions that are highly situated.

Despite surgical optimization, neoadjuvant treatments have an essential role in reducing the risk of local recurrence. Preoperative radiotherapy is still of interest for lower rectal tumors because it probably increases the chances of obtaining a healthy lateral resection margin, by partially treating the lesion [31]. It is preferred to postoperative radiotherapy because of better compliance, lower toxicity and greater efficacy on local control [32]. Chemotherapy delivered concomitantly with radiotherapy significantly reduces the risk of local recurrence, and is mainly intended for patients with lesions that are either borderline resectable or low-lying with a hope of conservative surgery in case of a decrease in tumor volume and lymph node extension [33]. Chemotherapy alone is only of proven interest as a palliative measure in inoperable and already irradiated locoregional relapses or in metastatic stages [34].

Immediate postoperative complications are essentially represented by urological complications such as urinary retention and ureteral wounds, pelvi-perineal infections and anastomotic fistulas, in addition to the complications inherent in all abdominal surgeries (respiratory complications, embolisms …). Late complications are mainly related to definitive colostomies (stenosis, prolapse, eventration) and to

lower colorectal and colo-anal anastomoses (increase in the number of stools, fragmentation of stools with frequent close exoneration, stool impaction, and even continence disorders) [37].

Other complications are sexual disorders (loss of sexual desire and dyspareunia due to vaginal dryness) and alteration of fertility, consequences of the surgical procedure as well as chemotherapy and radiotherapy.

Half of the patients treated for rectal cancer will die of a progressive recurrence. This may be due to locoregional recurrence or metastasis or a combination of both [24].

Several studies have reported the poor prognosis of rectal cancer in the young compared to the elderly with a 5-year survival between 9% and 50% [35,36].

## 4 Conclusion

Detection of patients with precancerous conditions, screening for cancer in subjects at risk (familial recto-colic cancer, familial recto-colonic polyposis and ulcerative colitis), suspicion of cancer in the presence of any proctological sign, early diagnosis and curative surgical resection preceded by radiotherapy are the means that can improve the prognosis of rectal cancer in young women.

## Ethical approval

I declare on my honor that the ethical approval has been exempted by my establishment.

## Sources of funding

None.

## Author contribution

Amal hajri: writing the paper and operating surgeon.

Amine Fatine: writing the paper and operating surgeon.

Eddaoudi Yassine: Corresponding author writing the paper.

Saad Rifki El Jay: study concept.

Rachid boufettal: study concept.

Driss erreguibi: study concept.

Farid Chehab: correction of the paper.

## Registration of research studies

researchregistry2464.

## Guarantor

DR FAT AM.

## Consent

Written informed consent for publication of their clinical details and/or clinical images was obtained from the patient.

This case series is compliant with the PROCESS Guidelines [38].

## Consent written

Informed consent was obtained from the patient for publication of this case report and accompanying images. A copy of the written consent is available for review by the editor-in-chief of this journal on request.

## Ethical approval

As per international standard written ethical approval has been collected and preserved by the author(s).

## Provenance and peer review

Not commissioned, externally peer-reviewed.

## Declaration of competing interest

The authors declare having no conflicts of interest for this article.
